# Long-term evolution of calcific tendinitis of the rotator cuff: clinical and radiological evaluation 10 years after diagnosis

**DOI:** 10.1186/s10195-021-00604-9

**Published:** 2021-10-26

**Authors:** Riccardo Compagnoni, Alessandra Menon, Simone Radaelli, Francesco Lanzani, Mauro B. Gallazzi, Alberto Tassi, Pietro S. Randelli

**Affiliations:** 1 Laboratory of Applied Biomechanics, Department of Biomedical Sciences for Health, Università degli Studi di Milano, Via Mangiagalli 31, 20133 Milan, Italy; 2 U.O.C. 1a Clinica Ortopedica, Azienda Socio Sanitaria Territoriale Centro Specialistico Ortopedico Traumatologico Gaetano Pini-CTO, Piazza Cardinal Ferrari 1, 20122 Milan, Italy; 3 U.O.C. Week Surgery di Ortopedia e Traumatologia, Azienda Socio Sanitaria Territoriale Centro Specialistico Ortopedico Traumatologico Gaetano Pini-CTO, Piazza Cardinal Ferrari 1, 20122 Milan, Italy; 4 U.O.C. Radiodiagnostica, Azienda Socio Sanitaria Territoriale Centro Specialistico Ortopedico Traumatologico Gaetano Pini-CTO, Piazza Cardinal Ferrari 1, 20122 Milan, Italy

**Keywords:** Shoulder, Arthroscopy, Calcific tendinitis, Rotator cuff, Long-term follow-up

## Abstract

**Background:**

Calcific tendinitis of the shoulder has a tendon involvement that could evolve to rotator cuff tear and shoulder osteoarthritis. This study aimed to evaluate the prevalence of glenohumeral osteoarthritis and rotator cuff tears in patients affected by calcific tendinitis at a minimum follow-up of 10 years after diagnosis.

**Methods:**

Patients diagnosed with calcific tendinitis of the shoulder with a minimum follow-up of 10 years were contacted and invited for a clinical and radiological evaluation. Information on the demographics, affected and dominant side, bilateral shoulder pain, type of treatment, habits, systemic or musculoskeletal diseases, reoperation of the index shoulder, and subjective satisfaction was collected. The clinical evaluation was performed using Constant–Murley score (CMS), American Shoulder and Elbow Surgeons Score (ASES), and numerical rating scale (NRS); isometric strength in forwarding flexion and abduction was also measured. Each patient also underwent an ultrasound examination to evaluate rotator cuff tendon integrity and a shoulder radiograph to evaluate osteoarthritis.

**Results:**

Seventy-nine patients were available for a phone interview, and 35 agreed to be examined. The mean age was 58.89 (± 7.9) years at follow-up. The prevalence of glenohumeral osteoarthritis was 17.14% in the study population, with significant progression in 14.29% of the cases, without rotator cuff full-thickness tears. x-Ray examination showed residual calcifications in 31 patients, with a mean diameter of 5.54 mm. In 30 cases, there was a reduction of the diameter; in 4 cases, the calcification increased in size; and in 1 case, the size did not change. The mean ASES score was 74.1 (± 22.7) in the group with calcifications larger than 2 mm and 89.4 (± 8.2) in patients with smaller calcifications (*p* = 0.08) without correlation with the type of treatment performed.

**Conclusions:**

Calcific tendinitis is a self-resolving disease without rotator cuff tears at long-term follow-up or degenerative glenohumeral progression.

Level of Evidence: 3, cohort study.

## Introduction

Calcific tendinitis, described by Duplay in 1872, is a common cause of severe shoulder pain, especially in the middle-aged female population, with patients typically reporting a sudden atraumatic limitation of range of motion. It is characterized by the presence of calcific deposits within the tendons of the rotator cuff and in the subacromial bursa, mainly consisting of calcium hydroxyapatite in crystalline or amorphous form [1–6]. Many hypotheses have been proposed to describe the etiology of calcific tendinitis over the years, without definitive conclusions. According to Uhthoff [3], the pathology is due to low blood perfusion that leads to metaplastic transformation of tenocytes into chondrocytes (chondrocite-like cells) with the production and deposition of calcium hydroxyapatite. He proposed that the development of the pathology consisted of three phases:

1. Precalcific: fibrocartilaginous metaplasia begins and is rarely symptomatic. In this phase the patient could be asymptomatic or complain of chronic pain that does not affect the function of the shoulder.
2. Calcific: divided into a formative phase, in which the deposition of calcium within the tendons occurs, and a reabsorption phase characterized by cell proliferation and colliquation of the calcified material. This leads to chemical irritation and increased intratendinous pressure, with development of shoulder pain that can be highly disabling and unresponsive to common analgesics.
3. Postcalcific: characterized by tendon tissue remodeling in which calcium deposits are replaced by granulation tissue and mature and vascularized scar tissue. This phase is characterized by subacute symptoms appearing as transient intensification of chronic symptoms [3, 7]

Calcific tendinitis is commonly considered a self-limiting disorder, with symptom relief usually obtained within a few weeks; however, hydroxyapatite deposits could remain for many months, or maybe years, within tendon tissue [8, 9], potentially causing rotator cuff tears and subsequent osteoarthritic evolution of the joint [10, 11].

Standard shoulder anteroposterior and axillary radiographs are useful to identify calcifications located within the soft tissues around the humeral head and in the subacromial space, providing the clinician with information about the location and morphology of the deposits. While the calcifications of the supraspinatus are clearly identifiable, those of the subscapularis or infraspinatus may be hidden due to the overlap of the humeral head.

Deposits were classified into four groups [12]:

- Well defined, dense, and homogeneous
- Well defined, dense, but fragmented
- Heterogeneous constituents of deposits with poorly defined contours
- Localized dystrophy at the tendon insertion

Types C and D are associated with the resorption phase, in which deposits are less clearly visible on radiography.

Ultrasound has become a reliable technique for diagnosing calcific tendinopathy of the shoulder, allowing the identification and localization of even small calcifications that appear to be hyperechoic with or without posterior acoustic shadow. It also allows evaluation of the integrity of the rotator cuff and the long head of the biceps, enabling a dynamic evaluation to be carried out, and is used for treatment with percutaneous needling.

Many different techniques have been proposed to reduce symptoms, from conservative treatments such as extracorporeal shock wave therapy (ESWT), to more invasive procedures such as ultrasound-guided needling or arthroscopic removal, without any definitive conclusions being drawn on the real usefulness of removing calcium deposits [13–17]. Only a few studies have analyzed the impact of residual calcific deposits on the rotator cuff tendon quality or its potential influence as a risk factor for glenohumeral osteoarthritis at long-term follow-up [18–20].

Therefore, the aim of this prospective clinical trial was to evaluate the prevalence of glenohumeral osteoarthritis and rotator cuff tears in a historical cohort of patients affected by calcific tendinitis, at least 10 years after the diagnosis, and to present the long-term clinical results associated with its management.

The primary aim of this study is to assess the prevalence of glenohumeral osteoarthritis in patients affected by calcific tendinitis at a minimum follow-up of 10 years after the diagnosis using x-ray evaluation.

Secondary outcomes were to evaluate the prevalence of rotator cuff tears through ultrasound examination and to assess shoulder pain and function using the Constant–Murley Score (CMS) the American Shoulder and Elbow Surgeons Score (ASES) and a numerical rating scale (NRS) [21–23].

## Materials and methods

The study protocol was approved by the Regional Ethical Committee (authorization number: Fondazione IRCCS Ca’ Granda Ospedale Maggiore Policlinico-Milano Area 2, Lombardia, Milan; 512/2017, October 31, 2017).

A single researcher performed an internal database search in November 2017 to identify all patients who referred to the emergency department between August 2001 and June 2008 with the clinical and radiographic diagnosis of calcific tendinitis.

The patients were identified using the specific numeric code (ICD-9 code 726.11) used in the emergency setting to classify this pathology.

Patients with clinical reports of calcific tendinitis confirmed by x-ray, age at diagnosis from 30 to 60 years, and follow-up of 10 years or more, were included in the study. Patients were excluded when the x-rays were not available, or if the images available did not support the diagnosis of calcific tendinitis.

Patients were contacted by phone and invited in the hospital to perform a clinical and radiological evaluation of the affected shoulder. All patients gave informed consent for each procedure.

The following data were collected: affected side, dominant side, age, gender, date of diagnosis, age at diagnosis, body mass index (BMI) at follow-up, smoking, type of job (light or hard physical demand), type of treatment [shock waves therapy and/or subacromial corticosteroids injections, conservative treatment such as physical therapy or nonsteroidal antiinflammatory drugs (NSAIDs)], diabetes, bilateral shoulder pain, inflammatory diseases, and other systemic or musculoskeletal diseases.

During the clinical evaluation, the NRS, ASES, and CMS were collected and isometric strength in shoulder forward flexion and abduction was measured.

All measures were performed in triplicate with a dynamometer (Kern HCB, Kern & Sohn GmbH, Germany) between October 2017 and October 2018.

A shoulder radiograph (standard true anteroposterior and lateral views) and an ultrasound examination were performed in all included patients. The ultrasound examination was executed by a dedicated musculoskeletal radiologist (M.B.G.) and used to define rotator cuff tendons integrity with Hinsley classification [24]. This classification considers four grades of lesion: (a) normal tendon, (b) abnormal tendon without partial or full-thickness tears, (c) partial thickness tear, and (d) full-thickness tear (subgrouped into longitudinal tear < 2.5 cm, transverse tear > 2.5 cm, and large full-thickness tear > 2.5 cm).

**Fig. 1:**
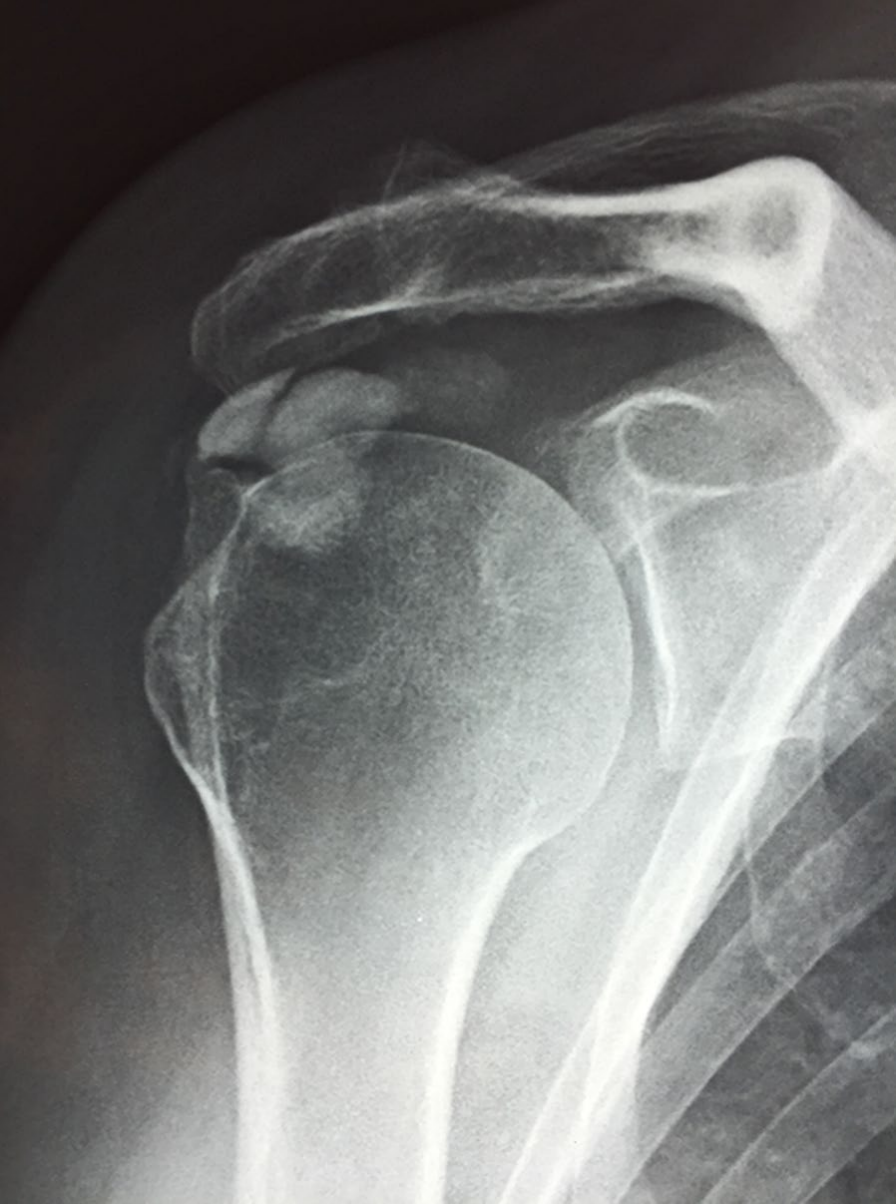
Residual calcification (Gärtner type 1). This patient was clinically and radiographically assessed 10  years after diagnosis. The x-ray showed the absence of arthritic signs

Shoulder osteoarthritis was classified on standard anteroposterior x-rays according to Samilson–Prieto classification [25], and the calcium deposits were measured on x-ray with IMPAX 6.5.2 and distinguished by morphology and density using the Gärtner classification [14], in both baseline and final control (Fig. 1).

Possible relationships between evidence of glenohumeral osteoarthritis progression and the following variables were investigated: (a) size of calcifications, (b) type of calcifications according to the Gärtner classification, (c) presence of rotator cuff lesions, and (d) specific treatment performed.

### Statistical analyses

Statistical analyses (A.M.) were performed with GraphPad Prism software (v 6.0; GraphPad Software Inc.) and SAS software (v 9.4; SAS Institute Inc.).

The differences between the groups of patients for continuous variables were evaluated with the unpaired Student’s *t*-test or Mann–Whitney *U* test according to the characteristics of the data distribution. Categorical variables were evaluated with the chi-square test or Fisher’s exact test. For all analyses, the significance level was set at *p* < 0.05.

## Results

### Baseline demographics

**Fig. 2:**
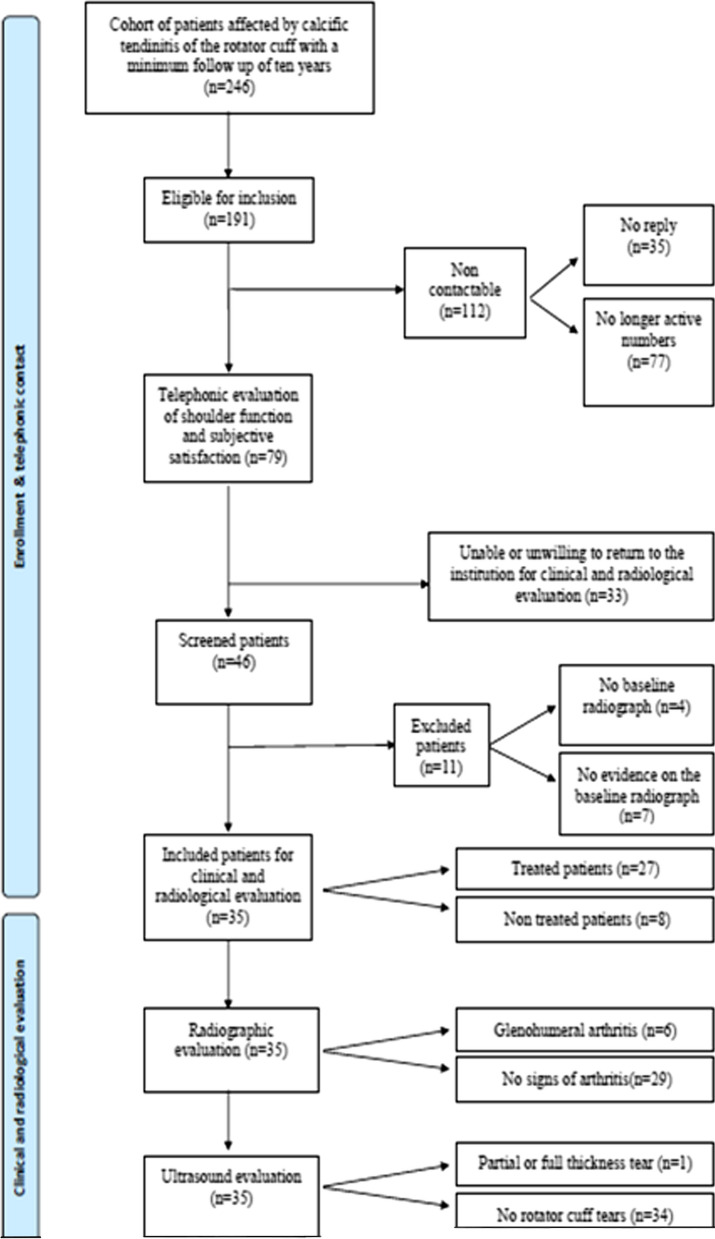
Flowchart of the study

One hundred ninety-one patients were identified and contacted using the numbers available in the internal database. Seventy-nine patients were available for a telephone interview, and 46 patients also agreed to return to the hospital for clinical and radiological evaluation. Eleven patients were further excluded because of the lack of baseline radiographs. Thirty-five patients were finally included in the analysis. A flow diagram illustrates the grouping and flow of patients in this clinical study (Fig. 2). Demographic data of the group of patients who received a clinical and radiographic assessment are reported in Table 1.

**Table 1:** Patient demographics

Variables	Overall
Age at follow-up (years)	58.89 (± 7.907)
Gender (F/M ratio)	0.74/0.26
BMI (kg/m^2^)	24.09 (22.15–25.28)
Follow-up (years)	13.00 (12.00–15.00)
Dominant side (L/R) ratio	0.14/0.86
Affected side (L/R) ratio	0.40/0.60
Smoker at follow-up (Y/N ratio)	0.31/0.69
Smoker at diagnosis (Y/N ratio)	0.40/0.60
Diabetes (Y/N ratio)	0.03/0.97
Job (light/hard)	0.60/0.40
Autoimmune disease (Y/N ratio)	0.25/0.75

Data are reported as mean (± SD), median (Q1–Q3), or frequency/ratio

*BMI* body mass index, *F/M* females/males, *N* No, *L/R* left/right, *Q1* first quartile, *Q3* third quartile, *SD* standard deviation, *Y* yes

Seventy-four percent of included patients were women, and the mean age was 58.89 (± 7.9) years at final follow-up of 13 years.

Calcifications afflicted the dominant side in 21 cases compared with 14 cases in the non-dominant side.

Regarding clinical comorbidities, the prevalence of diabetes was 3% and autoimmune diseases (rheumatoid arthritis, psoriatic arthritis, Hashimoto’s thyroiditis, diabetes mellitus type 1 (DM1), celiac disease, and psoriasis) were 25%.

Eleven patients smoked at follow-up, and 14 at baseline.

Regarding the medication administered, 7 patients were treated with corticosteroids injection, 8 subjects underwent shock waves therapy, 11 were treated with both corticosteroid injections and shock waves, and 1 patient was treated with ultrasound-guided percutaneous lavage.

Eight patients were treated with oral painkillers and antiinflammatories or physical therapy.

### Glenohumeral osteoarthritis

At baseline, radiographic signs of glenohumeral osteoarthritis were found in four patients (grade 1 Samilson–Prieto classification).

At final follow-up, the prevalence of glenohumeral osteoarthritis was 17.14% (six patients).

Osteoarthritis progression, defined as an increase of at least one grade in the Samilson–Prieto scale from baseline to follow-up, was found in 14.28% of the patients reviewed (five patients). An ex novo appearance of glenohumeral osteoarthritis was found in two patients, and an increase of one grade in the Samilson–Prieto classification was found in three patients.

### Subgroup analysis: glenohumeral osteoarthritis

The study population was further divided into two different groups according to the glenohumeral osteoarthritis progression as classified by Samilson and Prieto: patients with arthritic changes and patients without arthritic changes.

Stratified analysis was performed comparing demographic and clinical data to find potential risk factors for arthritic evolution (Tables 2, 3).

**Table 2:** Subgroup analysis: arthritic progression and patient characteristics

Group	Arthritic progression	No arthritic progression	*p*-Value
Age at follow-up (years)	58.3 (± 8.052)	62.4 (± 6.58)	0.4417 (n.s.)
Gender (F/M ratio)	0.80/0.20	0.40/0.60	0.0946 (n.s.)
BMI (kg/m^2^)	24.09 (22.15–25.28)	23.71 (22.27–25.46)	0.9545 (n.s.)
Follow-up (years)	12.00 (12.00–15.25)	14 (12.50–15.50)	0.6928 (n.s.)
Dominant side (L/R ratio)	0.10/0.90	0.40/0.60	0.2379 (n.s.)
Affected side (L/R ratio)	0.40/0.60	0.40/0.60	1.0000 (n.s.)
Smoker at follow-up (Y/N ratio)	0.33/0.67	0.20/0.80	1.0000 (n.s.)
Smoker at diagnosis (Y/N ratio)	0.37/0.63	0.60/0.40	0.3691 (n.s.)
Diabetes (Y/N ratio)	0.03/0.97	0.00/1	1.0000 (n.s.)
Job (light/hard)	0.57/0.43	0.80/0.20	0.6272 (n.s.)
Autoimmune disease (Y/N ratio)	0.23/0.77	0.40/0.60	0.5908 (n.s.)

Data are reported as mean (± SD), median (Q1–Q3), or frequency/ratio

*BMI* body mass index, *F/M* females/males, *N* No, n.s. not significant, *L/R* left/right, *Q1* first quartile, *Q3* third quartile, *SD* standard deviation, *Y* yes

**Table 3:** Subgroup analysis: arthritic progression and clinical and radiological results

	Overall	Arthritic progression	No arthritic progression	*p*-Value
Treatment (Y/N ratio)	0.77/0.23	0.80/0.20	0.60/0.40	0.5675 (n.s.)
Calcification at follow-up (Y/N ratio)	0.89/0.11	0.90/0.10	0.80/0.20	0.4766 (n.s.)
Calcification at follow-up > 5 mm (Y/N ratio)	0.34/0.66	0.40/0.60	0/1	0.1412 (n.s.)
Calcification diameter at diagnosis (mm)	12 (8–19)	12 (8–20)	12 (9–17.50)	0.9540 (n.s.)
Calcification diameter at follow-up (mm)	5 (2–6)	5 (3–7)	2 (2–4)	0.0692 (n.s.)
Variation of the calcification diameter (mm)	8.03 (± 7.71)	7.67 (± 8.01)	10.2 (± 5.72)	0.4131 (n.s.)
Gärtner classification (1/ > 1)	0.17/0.83	0.20/0.80	0/1	0.5610 (n.s.)
Partial or full-thickness RC tear (Y/N ratio)	0.03/0.97	0/1	0.20/0.80	0.1429 (n.s.)
Tendon degeneration (Y/N ratio)	0.31/0.69	0.30/0.70	0.40/0.60	0.6399 (n.s.)

Data are reported as mean (± SD), median (Q1–Q3), or frequency/ratio

n.s. not significant, *Q1* first quartile, *Q3* third quartile, *RC* rotator cuff, *SD* standard deviation, *Y* yes, *N* No

This analysis revealed that samples were homogeneous for all the considered parameters, no statistically significant differences were found for age, sex, BMI, follow-up, affected side, dominant side, smoking, type of job, diabetes, or autoimmune diseases (Tables 2, 3).

(Tables 2, 3).

No statistically significant association was found between glenohumeral osteoarthritis progression and the evaluated variables.

Although patients with arthritic changes showed a greater mean reduction of calcification diameter compared with non-arthritic patients (10.2 mm versus 7.7 mm, respectively), the differences in means between the two groups were not statistically significant.

On ultrasound evaluation, patients with osteoarthritis progression showed a higher percentage of degenerative manifestations on rotator cuff tendons, but the difference was not statistically significant.

No full-thickness tears of the rotator cuff were found at the final follow-up.

Given the extreme treatment variability, patients were subsequently divided in two groups (treated: corticosteroid injections and/or shock wave therapy and percutaneous lavage; untreated: physical therapy, NSAIDs, or nothing), and their clinical and radiological data were compared.

The mean variation of the calcification maximum diameter was 8.74 mm in the treated group compared with 5.63 mm in the untreated group, but no significant differences were noted (Table 4).

**Table 4:** Subgroup analysis: type of treatment and clinical and radiological results

	Treated (27 patients)	Untreated (8 patients)	*p*-Value
ASES: 0–100 (points)	76.1(± 20.1)	82.7(± 25.78)	0.2086 (n.s.)
NRS: 0–10 (points)	2.5(± 2.5)	1.6(± 2.6)	0.2181 (n.s.)
CMS: 0–100 (points)	76.3(± 7.3)	80.6(± 14.8)	0.1571 (n.s.)
Variation of the calcification (mm)	8.74 (± 7.47)	5.63(± 8.55)	1.0000 (n.s.)
Tendon degeneration (Y/N ratio)	0.33/0.67	0.25/0.75	1.0000 (n.s.)
Subacromial bursitis (Y/N ratio)	0.19/0.81	0.12/0.88	1.0000 (n.s.)
Calcification at follow-up (Y/N ratio)	0.85/0.15	1/0	1.0000 (n.s.)
Calcification at follow-up > 2 mm (Y/N ratio)	0.74/0.26	0.75/0.25	1.0000 (n.s.)
Calcification at follow-up > 5 mm (Y/N ratio)	0.33/0.67	0.37/0.63	1.0000 (n.s.)
Calcification at follow-up > 10 mm (Y/N ratio)	0.11/0.89	0.13/0.87	1.0000 (n.s.)

Data expressed as mean (± SD) or frequency/ratio

*ASES* American Shoulder and Elbow Surgeons Score, *CMS* Constant–Murley score, *mm* millimeters, *NRS* numeric rating scale, n.s. not significant, *SD* standard deviation, *Y* yes, *N* no

### Calcifications

At diagnosis, the calcifications had a diameter of 5–27 mm (average diameter 12.9 mm). The most affected tendon was the supraspinatus, followed by the subscapularis. Residual calcifications were observed in 31 patients (88.57%), with a diameter of 2–20 mm (average diameter 5.54 mm). At final follow-up, the residual calcifications in 8 cases had a diameter of less than 2 mm, 13 cases had a diameter of 3–9 mm, and 4 cases had a diameter greater than 10 mm.

**Fig. 3:**
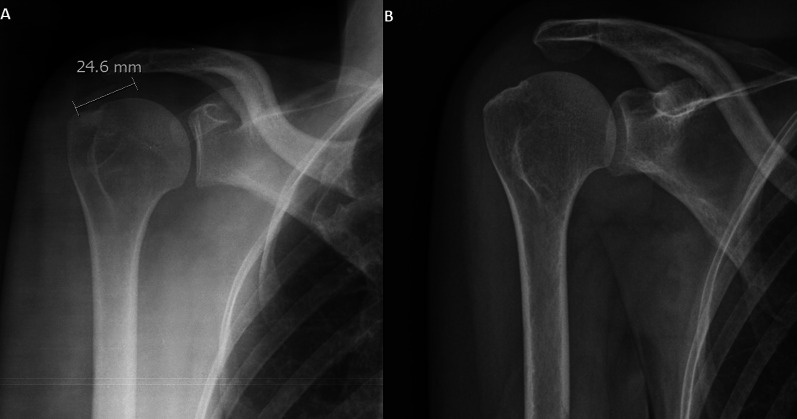
Anteroposterior shoulder x-ray performed at baseline (**A**) and after 10 years (**B**) shows complete resorption of the calcification

**Fig. 4:**
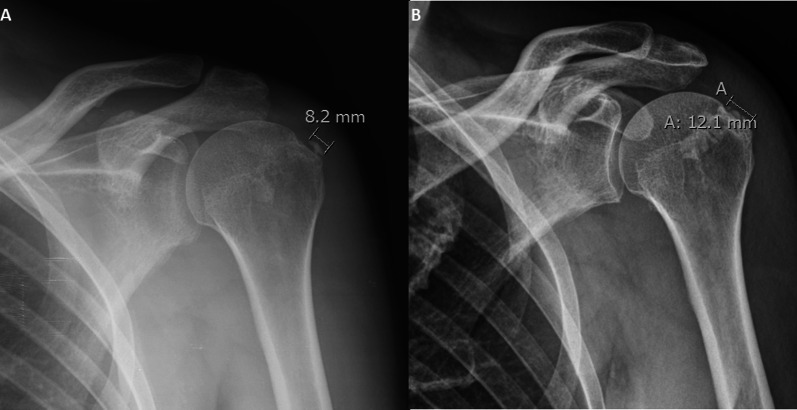
Anteroposterior shoulder x-ray performed at baseline (**A**) and after 10 years (**B**) shows the increased size of the calcification

**Fig. 5:**
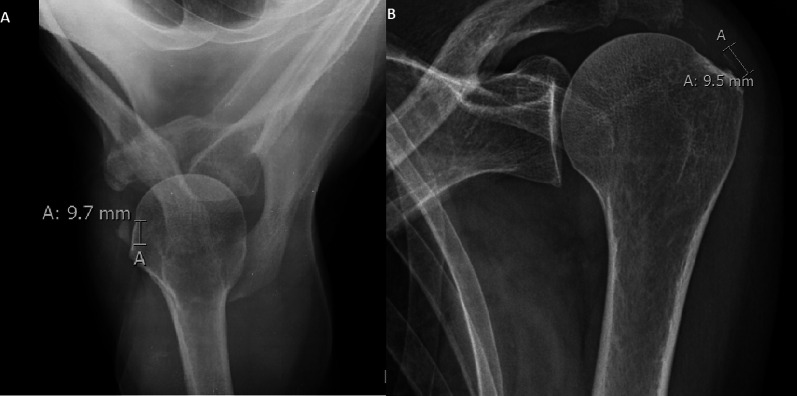
Anteroposterior shoulder x-ray performed at baseline (**A**) and after 10 years (**B**) shows the same length of calcification at follow-up

In 30 cases, a reduction of the diameter was found (mean reabsorption 57%), in 4 cases the calcification increased in size, and in 1 case the size did not change (Fig. 3, Fig. 4, Fig. 5).

### Subgroup analysis: persistence of calcifications

Calcifications with a maximum diameter ≤ 2 mm were compared with those ≥ 2 mm in diameter for the following variables. The mean ASES score was 74.1 (± 22.7) in the group with larger calcifications and 89.4 (± 8.2) in patients with smaller calcifications (*p* = 0.08). The NRS was 2.7 (± 2.7) in the larger calcification group compared with 1.0 (± 1.2) in the smaller calcification group (*p* = 0.22) (Table 5).

**Table 5:** Subgroup analysis: calcification diameter and clinical and radiological results

	Calcification at follow-up > 2 mm	Calcification at follow-up < 2 mm	*p*-value
ASES: 0–100 (points)	74.1(± 22.7)	89.4(± 8.2)	0.0813 (n.s.)
NRS: 0–10 (points)	2.7(± 2.7)	1.0(± 1.2)	0.2256 (n.s.)
CMS: 0–100 (points)	76.4(± 10.2)	80.2(± 6.12)	0.3372 (n.s.)
Treatment Y/N ratio	0.78/0.22	0.75/0.25	1.0000 (n.s.)
Subacromial bursitis Y/N ratio	0.18/0.82	0.12/0.88	1.0000 (n.s.)

Data are expressed as mean (± SD), median (Q1–Q3), or frequency/ratio

*ASES* American Shoulder and Elbow Surgeons Score, *CMS* Constant–Murley score, *NRS* numeric rating scale, *n.s.* not significant, *Q1* first quartile, *Q3* third quartile, *SD* standard deviation, *Y* yes, *N* no

## Discussion

The primary outcome of this study was the evaluation of the prevalence of glenohumeral osteoarthritis in patients affected by calcific tendinitis 10 years after diagnosis.

Radiological results did not show an increased prevalence of glenohumeral osteoarthritis in our patients.

A prevalence of glenohumeral osteoarthritis of 17.14%, similar to that estimated in the general Japanese population by Kobayashi et al. [26], was found. These authors observed a prevalence of glenohumeral osteoarthritis of about 15% in 541 subjects between 40 and 75 years old, demonstrating that patients with calcific tendinitis seem to have the same risk of osteoarthritic degeneration compared with the general population of the same age.

In this study, evaluation of rotator cuff tears using ultrasound did not identify full-thickness tears in any patient, but found signs of cuff degeneration in ten patients. This result is of great interest considering the potential damage that occurs over time from calcium deposits within tendon tissue.

Results of this study confirm the persistence of calcifications in the vast majority of the evaluated shoulders (88.57%), with a mean reabsorption of 57%. This is a relevant finding in contrast to the belief that the natural history of this pathology leads to full reabsorption of the deposits [3].

Better outcomes were reported in the four patients without calcifications compared with those who had residual deposits, and their dimension negatively influenced the outcome. Patients with radiological improvement over time have shown better clinical outcomes [19].

A hypothesis for this could be that the presence of calcific deposits alters shoulder biology, leading to inflammation as synovitis and subacromial bursitis, influencing shoulder kinematics and causing a slower or incomplete recovery.

Indeed, patients with smaller calcifications (diameter < 2 mm) showed better functionality of the shoulder during clinical evaluation and lower inflammatory signs on ultrasound images than those with larger ones, but no significant difference between the groups was recorded.

These findings are in agreement with those reported by other authors [19, 27], where a close association between better clinical outcomes and absence of calcifications at long-term follow-up was observed.

Porcellini et al. [27] reported similar results evaluating, clinically and with ultrasound, 63 patients affected by calcific tendinitis and treated by arthroscopic needling with a follow-up of 3 years. They found the persistence of calcifications in 45 patients who presented with lower mean clinical scores, and ascertained that the outcome was inversely related to the number and size of residual calcifications.

In the present cohort of patients, no statistically significant difference in terms of pain and functionality was found between patients subjected to conservative treatment and those who underwent corticosteroid injections and shock wave therapy.

The latter group had only slightly more reabsorption of the calcification compared with untreated (66.9% of the original deposit in the treated versus 59.3% in the untreated).

Results regarding pain relief and recovery are in line with those obtained by De Witte et al. [18], who assessed the evolution of calcific tendinitis with a long-term follow-up, and whose patients reached lower outcomes regardless of the treatment performed.

Regarding demographic characteristics, an increased prevalence of diabetes was not observed, whereas an association between these two pathologies has been reported [28].

The high prevalence of autoimmune diseases of about 25% in the included patients and an increased number of smokers compared with the general population (40%) are relevant data, reported in a few previous manuscripts [29, 30]. This group of pathologies and habits could probably influence the vascularity of the tendons, leading to an increase of calcium deposits and a reduction in tendon healing.

The present study has some limitations: first, the lack of a control group can give only partial information regarding the osteoarthritic evolution of the joint over the years. The prevalence of glenohumeral osteoarthritis, as well as that of rotator cuff tears, increases physiologically over time and the influence of calcific tendinopathy cannot be demonstrated without a control group.

Furthermore, there is a lack of literature data regarding the prevalence of rotator cuff tears in a population of comparable age with our patients, thus we cannot compare our findings with other data. However, the data obtained for rotator cuff tears is interesting, considering that no cases of complete rotator cuff tears were found in a population with an average age of 58 years.

The study design (prospective clinical trial on a historical cohort) did not allow inclusion of a clinical and ultrasound assessment at diagnosis that would have led to a more accurate comparison with the follow-up evaluation.

Furthermore, included patients have been subjected to many different treatments over a long period (10 years). To obtain significant results, the authors divided the patients into two groups, comparing those who underwent conservative treatment and those who were subjected to corticosteroid injections, shock waves therapy, or needling. These groups include different treatments, and selection bias is present.

Another limitation of this study is the high number of patients unwilling or unable to return to our institution for the clinical and radiological evaluations; a study conducted with a larger sample population would probably have given more significant results. Therefore, conclusions must be drawn with caution, especially for subgroups that present a small patient cohort (i.e., arthritic progression group, untreated group).

## Conclusions

Results of this study did not show a significant prevalence of glenohumeral osteoarthritis in patients affected by calcific tendinitis of the shoulder at follow-up of more than 10 years, but tendon calcifications are present in the majority of the patients and could be the cause of the persistence of minor symptoms.

The lower clinical outcomes obtained by patients included in this study do not seem to be related to the presence of glenohumeral joint pathologies such as osteoarthritis or rotator cuff tears.

If the association between persistence of calcifications and long-term follow-up symptoms is confirmed, it would be interesting to see whether a faster and more aggressive approach, such as percutaneous lavage or arthroscopic needling, could give better results than the conservative treatments.

## Data Availability

Data and materials generated during the current study are available from the corresponding author on reasonable request.
